# Ertapenem in Hidradenitis Suppurativa: A Review of Antimicrobial Effects and a Proposed Immunomodulatory Role

**DOI:** 10.3390/antibiotics15100995

**Published:** 2026-10-08

**Authors:** Esther O. Nwozo, Rebecca Metellus, Beverly Udegbe, Robert J Kerns, Christopher G. Bunick, Michal Kidacki

**Affiliations:** 1 The Lewis Katz School of Medicine, Temple University, 3500 N. Broad Street, Philadelphia, PA 19140, USA; esther.nwozo@temple.edu (E.O.N.); beverly.udegbe@temple.edu (B.U.); 2 Geisinger Commonwealth School of Medicine, 525 Pine St., Scranton, PA 18509, USA; rmetellus@geisinger.edu; 3 College of Pharmacy, University of Iowa, 180 S Grand Ave, Iowa City, IA 52242, USA; robert-kerns@uiowa.edu; 4 Program in Translational Biomedicine, Department of Dermatology, Institute for Global Health, Yale University School of Medicine, 20 York Street, New Haven, CT 06518, USA; christopher.bunick@yale.edu; 5 Department of Dermatology, University of Iowa, 200 Hawkins Drive, Iowa City, IA 52242, USA

**Keywords:** acne inversa, antibiotic, carbapenems, inflammation, innate immunity, stewardship, anaerobic bacteria

## Abstract

Hidradenitis suppurativa (HS) is a chronic, inflammatory skin disorder characterized by recurrent nodules, abscesses, and sinus tract formation, driven by dysregulated innate immunity and microbial dysbiosis. Intravenous ertapenem has emerged as a therapeutic option for severe, refractory HS, with clinical studies consistently demonstrating meaningful improvements in pain, drainage, inflammatory markers, and quality of life. While these benefits have traditionally been attributed to ertapenem’s broad-spectrum antimicrobial activity against the polymicrobial anaerobic flora of HS lesions, the rapid onset of clinical improvement and inconsistent anaerobic suppression observed during treatment suggest that additional mechanisms may be at play. Emerging mechanistic evidence from studies of carbapenems in non-HS experimental systems suggests that this drug class may influence host inflammatory pathways through effects on cytokine signaling, endotoxin release, neutrophil and macrophage activation, and NF-κB inhibition. Additionally, ertapenem’s unusually high plasma protein binding, particularly to acute-phase reactants elevated in HS such as haptoglobin and fibrinogen, may further influence drug distribution and immune signaling within inflamed tissues. This review examines the antimicrobial and emerging immunomodulatory properties of ertapenem and explores how these convergent mechanisms may explain its clinical efficacy in severe HS, while highlighting stewardship considerations and directions for future research. **Methods:** A structured search of PubMed/MEDLINE was conducted from database inception through 1 May 2026 using terms related to hidradenitis suppurativa, ertapenem, carbapenems, antimicrobial activity, immunomodulation, inflammatory signaling, innate immune cells, and pharmacokinetics. During revision, a targeted literature update through 1 September 2026 was performed to incorporate newly published and reviewer-requested studies. Eligible clinical, microbiologic, pharmacologic, and mechanistic studies were screened by title, abstract, and full text and synthesized narratively.

## 1. Introduction

Carbapenems are a subclass of β-lactam antibiotics with broad-spectrum activity against Gram-negative, Gram-positive, and anaerobic organisms. Like other β-lactams, carbapenems exhibit antimicrobial activity by binding to penicillin-binding proteins and inhibiting bacterial cell wall transpeptidation. Disruption of this process results in bacterial lysis and cell death, making carbapenems bactericidal agents [1]. Beyond their antimicrobial activity, the biological consequences of bacterial killing may play an important role in host inflammation. Bacterial cell wall lysis can release immunologically active components such as lipopolysaccharide (LPS), which may activate downstream immune receptors and cytokine signaling pathways. Thus, antibiotic-mediated bacterial killing can also shape host immune signaling, as the mechanisms of bacterial lysis can influence host inflammatory responses [1].

Emerging evidence suggests that antibiotics may exert immunomodulatory effects independent of their antimicrobial activity. In an ex vivo human whole-blood model, exposure to different antibiotics significantly altered cytokine responses following LPS stimulation, demonstrating that antimicrobial agents can modify host inflammatory signaling even in the absence of active infection [2]. Antibiotic treatment influenced the magnitude of cytokine release, including key mediators such as tumor necrosis factor-α (TNF-α) and interleukin-10 [2]. Although these studies were conducted in systemic inflammation models, they suggest that antibiotics can influence inflammatory signaling in disease states beyond acute infection. The use of carbapenem to treat inflammatory skin conditions such as hidradenitis suppurativa (HS) provides real-world evidence of the immunomodulatory properties of this antibiotic class.

HS is a chronic inflammatory skin disorder characterized by recurrent painful nodules, abscesses, and sinus tract formation primarily affecting intertriginous areas. The estimated global prevalence ranges from approximately 0.00033% to 4.1%, with higher prevalence reported in Western populations [3,4]. Importantly, HS disproportionately affects individuals with skin of color, particularly African American populations, who experience higher prevalence and often greater disease severity compared with White populations [5]. These disparities contribute to delayed diagnosis, more advanced clinical presentations, and significant psychosocial impact. In addition to its cutaneous manifestations, HS is also associated with chronic pain, impaired quality of life, and multiple comorbidities, including metabolic and cardiovascular disease [4,5]. The pathogenesis of HS is complex and multifactorial, involving follicular hyperkeratosis, follicular rupture, and a sustained inflammatory response that drives chronic tissue remodeling [4]. Central to this process is dysregulation of the innate immune system, with increased activity of neutrophils and macrophages and increased expression of proinflammatory cytokines such as TNF-α, IL-1β, and IL-17A/F within HS lesions [6]. Alterations in the cutaneous microbiome also appear to play an important role in maintaining this inflammatory environment. Microbial dysbiosis, including shifts in commensal and pathogenic bacterial populations and formation of biofilms, may sustain immune activation and hinder inflammatory resolution [6].

The microbial landscape of HS lesions has been increasingly characterized through culture-based and next-generation sequencing (NGS) studies, revealing a pattern of dysbiosis distinct from healthy skin. Prospective metagenomic analysis of HS lesions identified 43 bacterial taxa associated with disease, with two Gram-negative anaerobic rods, *Prevotella* and *Porphyromonas*, predominating, while aerobic skin commensals were notably reduced in abundance [7]. Lesion samples were particularly enriched in anaerobic genera of the *Bacteroidetes* and *Fusobacteria* phyla, with *Prevotella*, *Porphyromonas*, and *Fusobacterium* representing the most abundant lesional taxa, contrasting with a diminished presence of normal skin commensals such as *Corynebacterium* and coagulase-negative staphylococci [7]. Complementary NGS data from follicular biopsy specimens corroborate these findings. Differential abundance analyses demonstrated a significantly increased relative abundance of *Porphyromonas* and *Peptoniphilus* species in HS lesional skin compared to healthy controls, while *Cutibacterium acnes* showed a significantly higher relative abundance in healthy controls, suggesting its depletion may contribute to HS pathogenesis [8]. Classical bacterial pathogens appear to play a limited role in this process. *Staphylococcus aureus* was associated with HS lesions but was never cultured as a predominant pathogen, and even in severe Hurley stage 3 disease, it represented only a minor proportion of lesional taxa [7]. However, reported anaerobic taxa and their relative abundance vary across studies, likely reflecting differences in sampling technique (i.e., superficial swab versus deep tissue biopsy), transport and culture conditions for oxygen-sensitive organisms, and the sequencing method used. These methodological differences could complicate direct comparison across cohorts and should be considered when interpreting the apparent consistency of anaerobic dominance in HS lesions. Furthermore, it is unclear if these organisms initiate inflammation or preferentially colonize the established inflammatory niches [9]. In this complex inflammatory environment, systemic antibiotics have been a cornerstone of HS therapy [10]. Notably, systemic antibiotics continue to be used even during biologic therapy; in a recent multicenter cohort of patients receiving adalimumab, repeated antibiotic courses were used for inflammatory flares, persistent lesions, or incomplete disease control [11]. Emerging evidence, however, suggests that their clinical benefit may extend beyond antimicrobial effects to include modulation of inflammatory signaling [12].

Among systemic antibiotics used in HS, ertapenem has been increasingly used as a therapeutic option for severe or refractory disease. Clinical studies have reported substantial improvements in inflammatory lesions, pain, and disease severity following ertapenem therapy, often used as a bridge to surgical intervention or other systemic treatments [13,14,15,16,17]. While these benefits may be at least partially attributed to antimicrobial effects, additional immunologic mechanisms may contribute to therapeutic response. Microbiologic findings in ertapenem-treated HS cohorts do not consistently reflect sustained anaerobic suppression. Taxa such as *Prevotella* have been observed to decline early in treatment but re-emerge during ongoing therapy, suggesting that clinical improvement may not be fully explained by antimicrobial activity alone [16]. In this review, we examine the emerging evidence supporting a potential immunomodulatory role for ertapenem in HS and explore how its antimicrobial and host-directed effects may converge to influence disease inflammation.

## 2. Clinical Use of Ertapenem in Hidradenitis Suppurativa

Intravenous ertapenem has been primarily utilized in patients with severe, refractory HS who have failed conventional medical management. The patient population across studies typically includes individuals with Hurley stage II and III disease, characterized by suppurating lesions, extensive anatomical involvement, and a decline in quality of life, for whom standard topical and systemic therapies have proven inadequate [18]. Early clinical experience with intravenous ertapenem included cohorts of patients with severe HS who were either not surgical candidates due to the extent of their HS lesions, declined surgical intervention, or required rapid improvements in their lesions prior to surgery [13]. Furthermore, North American clinical management guidelines categorize intravenous ertapenem as a rescue therapy used during surgical planning in patients with advanced HS [18]. Updated European S2k guidelines similarly include intravenous ertapenem as an option for severe HS, particularly for down-staging inflammatory disease before surgery, using a 1 g daily regimen for 6 weeks [19]. Although surgery remains one of the cornerstones of treatment for severe HS, ertapenem may temporarily reduce inflammation and suppuration, thereby improving patients’ preparedness for surgery. Beyond the bridge-to-surgery indication, this population was further characterized as those who continued to experience disease progression following prior antibiotic regimens and biologic therapies, reinforcing that ertapenem can be beneficial in the most resistant cases [16]. Collectively, the literature suggests the ideal candidate for intravenous ertapenem includes patients with Hurley stage II or III disease who require aggressive short-term medical management either as definitive therapy or as optimization prior to surgical intervention or initiation of biologic therapy.

Treatment regimens and duration of intravenous ertapenem for hidradenitis suppurativa vary, though it has generally been administered at a dose of 1 g IV daily in published reports. The foundational protocol consisted of a 6-week ertapenem induction course administered at home via a peripherally inserted central catheter (PICC) line, followed by a 6-week specialized oral regimen of rifampicin, moxifloxacin, and metronidazole [14]. This regimen was subsequently narrowed to rifampin and moxifloxacin alone for an additional six weeks to reduce the risk of neurologic adverse effects associated with prolonged metronidazole exposure. Patients achieving full clinical remission were then transitioned to secondary prophylaxis with co-trimoxazole, underscoring the importance of maintenance therapy in preventing relapse. Building on the 6-week protocol, Nosrati et al. extended the duration to 12–16 weeks while continuing adjunctive biologics and antiandrogens such as spironolactone, reporting that this prolonged course yielded more sustained improvements than shorter regimens [16]. Other studies employed greater flexibility in duration with ertapenem treatment courses ranging from 8 to 128 days and concurrent therapies including biologics, systemic antibiotics, spironolactone, and topical agents [13,14,15,17,20,21]. The use of ertapenem as an adjunctive therapy reflects real-world practice, in which ertapenem is used alongside additional therapies rather than in isolation. The North American guidelines acknowledge the various timelines, which opens the door to future studies to determine the optimal duration of ertapenem therapy, especially for patients with advanced and refractory HS. When selecting candidates for ertapenem therapy, it is important to consider the potential barriers to this care, including route of administration, the need for PICC line placement, and the risk of antimicrobial resistance [18]. Overall, the literature suggests that while 1 g IV daily is the established dose, optimal duration remains an unresolved variable, with emerging evidence favoring extended courses of 12–16 weeks over the original 6-week protocol for more durable disease control. New reports, however, indicate that ertapenem efficacy may be maintained even at lower doses of 250 mg every other day [20].

Across studies, intravenous ertapenem has consistently demonstrated meaningful improvements in pain, drainage, and overall quality of life in patients with severe HS [17,22]. Substantial reductions in patient-reported outcomes were observed following the 6-week induction course, with median pain scores falling from 6 to 0, drainage scores decreasing from 8 to 2, and daily handicap scores improving from 8 to 3 [14]. Building on these findings, Nosrati et al. reported improvements in Physician Global Assessment from a mean of 3.9 to 2.7 and a reduction in pain from 4.2 to 1.8 following a 12–16-week course of intravenous ertapenem. Within this 98-patient cohort, 80% reported medium to high satisfaction with treatment [16]. Despite varying approaches to the length of ertapenem therapy, especially for patients with Hurley stage II or III HS, ertapenem therapy is associated with widespread improvement in quality of life [13]. To contextualize the clinical significance of these findings, it is important to recognize that severe HS carries one of the highest quality-of-life burdens among chronic dermatological conditions [23]. This burden can present in a multitude of ways, including absenteeism from work and educational settings, social withdrawal, and psychological comorbidities. These outcomes are corroborated by patient-reported follow-up interviews, in which 85.7% of patients reported moderate or significant improvement in quality of life, 82.1% reported meaningful pain reduction, and 89.3% reported improved drainage [13]. As 75% of patients were willing to undergo treatment again, this demonstrates a favorable patient outcome despite the burden of IV therapy [13]. The reduction in drainage observed during ertapenem treatment may partly reflect its activity against the polymicrobial, predominantly anaerobic flora associated with chronic suppurating HS lesions, although this mechanism has not been directly demonstrated [7,14]. This outcome has direct implications for patients’ functional and social capacity, as chronic purulent discharge represents a major disabling and stigmatizing feature of severe HS. The North American clinical management guidelines further support these findings on a larger scale, as they note that patients with Hurley stage I and II underwent remission following ertapenem use, while patients with Hurley stage III HS reported great improvements in quality of life, even if they did not reach full clinical remission [14,18] (Table 1). All available studies are retrospective, single-center, and uncontrolled, with variable follow-up and heterogenity in ertapenem dose, duration, and concurrent therapy. Every reported cohort also included other treatments, such as biologics, other antibiotics, or surgery, making it difficult to attribute improvement to ertapenem alone rather than multimodal therapy.

A notable limitation of the ertapenem clinical trial findings is that Prevotella species, *Enterococcus faecalis*, and *Bacteroides ovatus* persisted despite active ertapenem therapy [16]. This observation introduces ambiguity regarding the pathogenic role of these organisms in HS. If these taxa were primary drivers of HS pathogenesis, as has been proposed in the literature, ertapenem, a broad-spectrum carbapenem with activity against anaerobic and Gram-negative organisms, would be expected to suppress their populations [7,25]. Conversely, their persistence under antibiotic pressure may suggest that these organisms represent commensal constituents of the cutaneous or lesional microbiome rather than obligate pathogens, thereby questioning their etiological significance in HS. Notably, ertapenem lacks reliable activity against Pseudomonas aeruginosa. While not a predominant organism in HS lesional microbiome studies, it has been detected in some analyses within chronic tunnels or draining sinuses [7,8,25]. This ambiguity underscores the methodological constraints inherent to current HS microbiome research. Culture-based approaches operating under aerobic laboratory conditions are susceptible to selection bias, potentially failing to capture fastidious or obligate anaerobic pathogens that require specialized growth conditions. Moreover, 16S rRNA amplicon sequencing, while widely utilized, offers limited phylogenetic resolution compared to shotgun metagenomic sequencing, rendering it insufficient for reliably discriminating between pathogenic and commensal species at the strain level.

Although clinical outcomes have often been observed in studies of ertapenem therapy, inflammatory markers frequently correlate with the disease severity of HS [26]. Following 12–16 weeks of ertapenem therapy, Nosrati et al. observed a reduction in C-reactive protein (CRP) from 5.5 to 2.4 mg/dL, IL-6 from 25.2 to 13.7, and leukocyte count from 11.3 to 10.0 [16]. Changes in inflammatory markers during ertapenem therapy are noteworthy but cannot distinguish direct immunomodulatory effects from reductions in microbial burden or overall disease activity. Collectively, these findings establish ertapenem as a pivotal therapy for achieving meaningful clinical reductions in the major symptoms associated with severe HS. Given the consistent improvements in quality of life, drainage, and pain across numerous studies, ertapenem is positioned to be an effective therapy in addressing HS disease burden.

## 3. Immunomodulatory Effects of Carbapenems: Mechanistic Insights

Clinical improvement following ertapenem therapy in severe HS often occurs rapidly, sometimes earlier than expected from bacterial eradication alone [14,17,24]. This observation raises the possibility that ertapenem exerts effects beyond antimicrobial activity, potentially influencing host inflammatory pathways. Such a hypothesis is biologically plausible given the inflammatory architecture of HS, which is characterized by dysregulated innate immune signaling, macrophage and neutrophil activation, and elevated pro-inflammatory cytokines including TNF-α, IL-1β, and IL-6 [6]. If carbapenems are capable of altering cytokine signaling or innate immune cell behavior independently of microbial clearance, their therapeutic benefit in HS may partially reflect modulation of these inflammatory processes. Consequently, mechanistic studies examining how carbapenems interact with immune signaling may provide a framework for understanding their clinical effects in HS.

Experimental evidence suggests that carbapenems can influence inflammatory cytokine production, although these effects appear highly context-dependent. In vitro experiments using human polymorphonuclear cells and monocytes found that meropenem did not substantially alter leukocyte viability or immune function at therapeutically relevant concentrations, although higher concentrations reduced LPS-stimulated TNF-α release and neutrophil superoxide production, suggesting selective effects on inflammatory signaling [27]. In a mechanistic study of Klebsiella pneumoniae infection, meropenem treatment reduced several pro-inflammatory mediators, including IL-1β, IL-6, and TNF-α, when bacterial clearance was achieved [28]. These findings suggest that, in some experimental settings, carbapenems may influence host inflammatory responses in addition to their antimicrobial effects. Notably, neutrophil levels declined even in carbapenem-resistant isolates where bacterial clearance was not achieved, suggesting that this immunomodulatory effect is independent of antimicrobial activity and particularly relevant to HS, where neutrophil activation is a central driver of disease [28].

Individual carbapenems may influence inflammatory signaling differently. In a murine pneumonia model, doripenem, meropenem, and imipenem produced distinct cytokine responses despite comparable antibacterial activity [29]. Doripenem treatment was associated with early pulmonary IL-6 induction and preservation of systemic IFN-γ signaling, whereas meropenem and imipenem were associated with reductions in circulating IFN-γ and greater endotoxin release during therapy [29]. These differences are thought to arise from variations in penicillin-binding protein affinities and bacterial killing dynamics, which can influence the quantity and timing of microbial component release during treatment. Because bacterial products such as endotoxin strongly activate innate immune pathways, antibiotic-specific differences in bacterial lysis may indirectly shape host cytokine responses. Therefore, inflammatory changes observed during carbapenem therapy may reflect differences in microbial clearance kinetics rather than direct immune modulation by the drugs themselves, highlighting the importance of separating antimicrobial effects from host-directed immunologic activity. These agent-specific differences also caution against extrapolating mechanistic findings from meropenem, imipenem, or doripenem directly to ertapenem. Accordingly, these studies support potential class-level mechanisms but do not establish that ertapenem produces the same immunologic effects in HS.

Beyond cytokine production, carbapenems may influence macrophage behavior and innate immune signaling. In a murine model of multidrug-resistant Klebsiella pneumoniae infection, carbapenem exposure, particularly imipenem, enhanced bacterial outer membrane vesicle (OMV) release and increased macrophage uptake of these vesicles through LOX-1-dependent mechanisms [30]. This process triggered caspase-11-mediated pyroptosis and increased production of IL-1β, IL-18, and TNF-α, demonstrating that antibiotic exposure can amplify inflammasome-driven inflammatory signaling even when bacterial burden remains unchanged [30]. Extension experiments showed that meropenem, ertapenem, and biapenem similarly increased GroEL-rich OMV release and mouse mortality, suggesting that aspects of this response may extend across the carbapenem class, although pyroptosis experiments were primarily characterized with imipenem [30].

Earlier studies suggest that carbapenems may also influence innate immune clearance mechanisms. In vitro and clinical observations showed that imipenem increased neutrophil phagocytic activity without significantly altering lymphocyte IL-2 signaling or soluble IL-2 receptor levels [31]. Complementary experimental work further indicates that β-lactam antibiotics can augment macrophage TNF production through enhanced release of bacterial structural components during microbial killing [32]. Together, these findings highlight how antibiotics may reshape macrophage responses by modifying both host immune cell behavior and the inflammatory signals generated during bacterial lysis.

At the level of intracellular signaling, carbapenems may also exert direct anti-inflammatory effects. A drug-repurposing screen identified imipenem as a suppressor of cytokine-storm signaling in macrophage and murine endotoxemia models, where it reduced multiple inflammatory mediators, including TNF-α, IL-6, and IL-1β, through inhibition of NF-κB activation [33]. Many of these cytokines, including TNF-α, IL-1β, and IL-6, are central mediators of HS inflammation, suggesting a potential mechanistic link between carbapenem-mediated immune modulation and clinical improvement observed in severe disease. The coexistence of pro-inflammatory and anti-inflammatory effects across different models suggests that carbapenem-mediated immune modulation is highly context-dependent. Consequently, how these macrophage-directed effects translate to chronic inflammatory diseases such as HS remains uncertain and warrants further mechanistic investigation.

**Figure 1:**
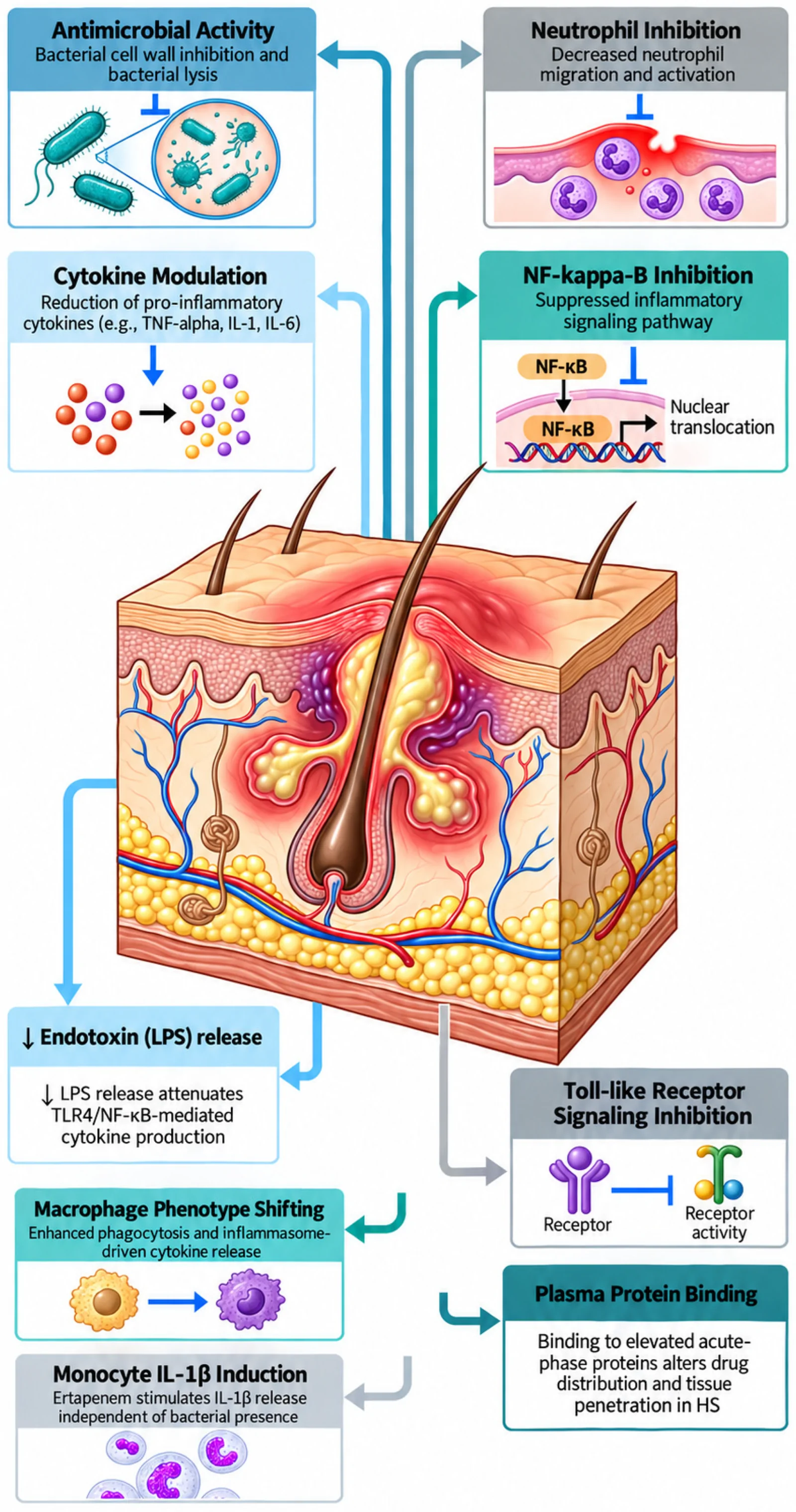
Proposed mechanisms of ertapenem activity in hidradenitis suppurativa.

Carbapenems may also influence inflammation indirectly by altering endotoxin release during bacterial killing. In vitro studies comparing antibiotic classes demonstrated that meropenem and imipenem induce significantly lower levels of LPS release from Escherichia coli than certain cephalosporins, particularly ceftazidime [34]. Because LPS is a potent activator of Toll-like receptor-4 signaling and downstream NF-κB-mediated cytokine production, differences in endotoxin liberation during antimicrobial therapy could substantially affect inflammatory signaling. Although these experiments were performed in bacterial culture systems without immune cells, they provide a mechanistic explanation for how antibiotic pharmacodynamics may shape host inflammatory responses independent of microbial clearance. Reduced endotoxin release during carbapenem therapy may therefore attenuate downstream inflammatory signaling. These findings suggest that differences in endotoxin release across antibiotic classes may represent an underrecognized factor of host inflammatory responses during antimicrobial therapy (Table 2; Figure 1).

Of these mechanisms, NF-kB-mediated suppression of macrophage cytokine output and reduced endotoxin release during bacterial killing are best supported across independent models, and both are consistent with the rapid clinical improvement seen with ertapenem. The proinflammatory findings come from acute infection models and may not translate to the chronic inflammation in HS. Human data corroborate this, as meropenem reduced expression in peripheral blood mononuclear cells from patients with acute bacterial infection, though the authors interpreted this as a feature of sepsis-induced immunosuppression rather than a therapeutic effect [28,35].

**Table 2:** Summary of Mechanistic Studies examining Immunomodulatory Effects of Carbapenems across Experimental Models.

Study	Cell Type Studied	Carbapenem Studied	Cytokines/Pathways Affected	Direction of Effect	Model System	Limitations
Yang [28]	Macrophages, neutrophils	Meropenem	IL-1β, IL-6, TNF-α, IL-27, CSF2/3, CCL5, CCL11, CXCL2/13, Socs3, Nos2, Ccr7; IFN-γ	Mixed: strong infection-driven inflammation; meropenem suppresses cytokines in CSKP but IL-6 remains elevated in CRKP	Murine infection model	Single capsule type (K144, ST11); murine immune context; limited generalizability to diverse CRKP
Hilliard [29]	Macrophages, neutrophils	Doripenem, meropenem, imipenem	Doripenem: ↑ early lung IL-6, preserved serum IFN-γ, ↑ IL-10 trend Meropenem/Imipenem: ↓ systemic IFN-γ, ↑ endotoxin release; IL-1β unaffected	Mixed, agent-specific	Murine lethal pneumonia model (acute LRTI)	Single ESBL strain; murine acute model; cytokine effects early and modest
Ye [30]	Macrophages	Imipenem	↑ IL-1β, IL-18, TNF-α via OMV-caspase-11 pyroptosis	↑ Pro-inflammatory	Murine MDR *K. pneumoniae* pneumonia	Murine only; limited cytokine panel
Pasqui [31]	Neutrophils, T cells, B cells	Imipenem	IL-2, sIL-2R unchanged; ↑ neutrophil phagocytosis	Neutral cytokine effect	Human in vitro assays and small clinical cohort	Small sample; narrow cytokine measurement; short follow-up
Su [33]	Macrophages; hPBMCs	Imipenem	↓ TNF-α, IL-6, IL-1β, IL-12, IL-17A, IL-27, MCP-1, GM-CSF; ↓ IFN-α/γ via NF-κB inhibition	↓ Broad anti-inflammatory	LPS-induced sterile endotoxemia (mouse and human cells)	Not infection-driven; pretreatment design
Trautmann [34]	None (bacterial culture only)	Meropenem, imipenem	↓ free and cell-associated LPS release vs. ceftazidime	Indirect anti-inflammatory (endotoxin reduction)	In vitro *E. coli* culture	No immune cells; supratherapeutic concentrations
Smelter [36]	Monocytes, T, B, NK cells	Ertapenem	↑ IL-1β (combination > single agent)	↑ Pro-inflammatory	Human PBMC in vitro	Single cytokine

## 4. Plasma Protein Binding of Ertapenem and Potential Immunologic Implications in HS

**Figure 2:**
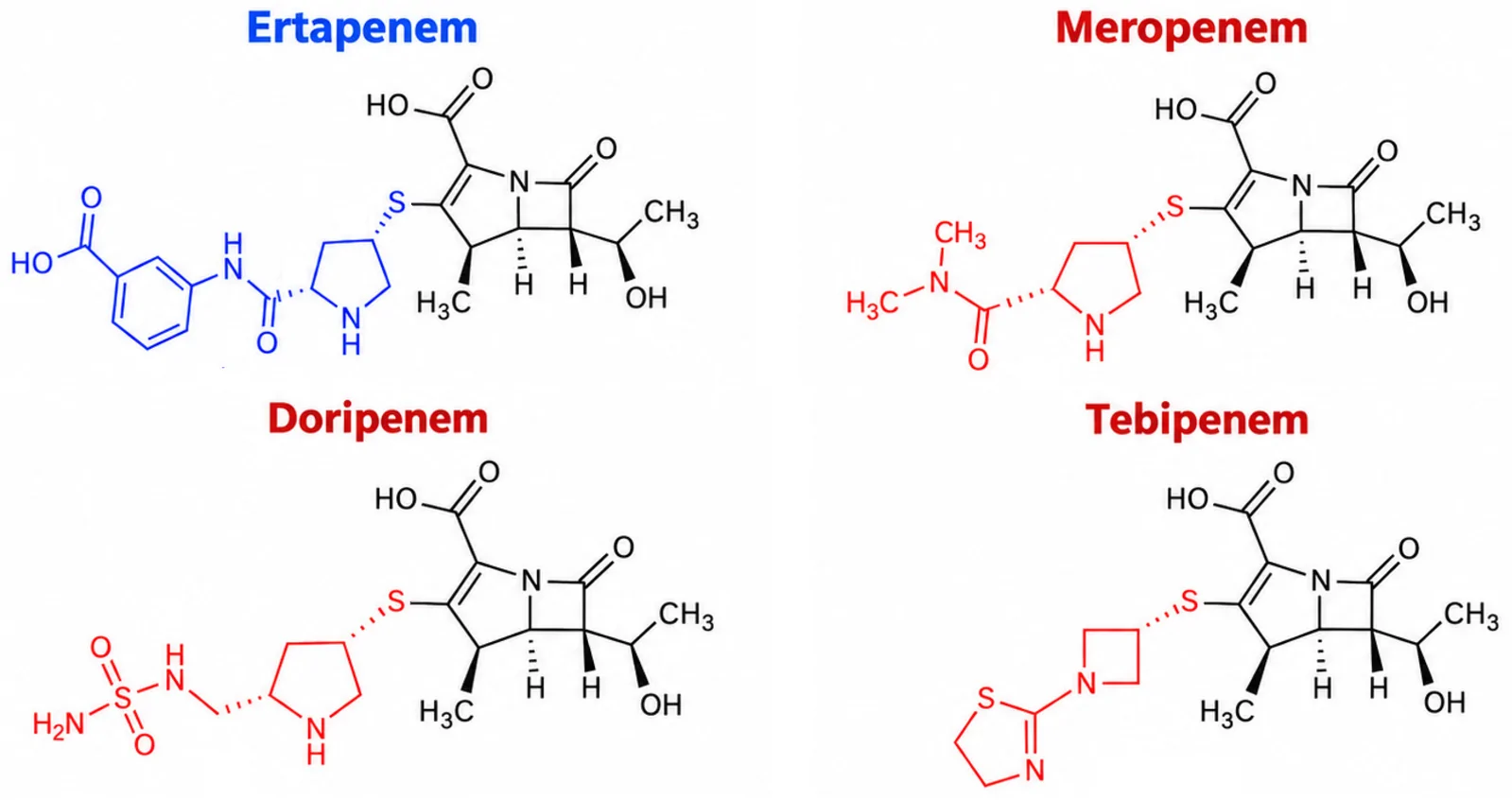
Structural Comparison of Ertapenem and Other Carbapenems. Common carbapenem core shown in black; differentiating side chains highlighted in color.

Due to its structure, ertapenem differs from other carbapenems in its unusually high plasma protein binding, with approximately 85–95% of circulating drug bound to serum proteins (Figure 2). Proteomic analyses have identified multiple ertapenem-binding partners beyond albumin, including fibrinogen, haptoglobin, immunoglobulin G, complement proteins, fibronectin, and α1-antitrypsin (Table 3) [37]. These interactions significantly reduce the free fraction of active drug in vitro and contribute to the prolonged half-life that distinguishes ertapenem pharmacokinetically from other carbapenems. These observations are experimentally established; however, their relevance to HS remains uncertain. Because several ertapenem-binding proteins are elevated during systemic inflammation and in patients with HS, we hypothesize that changes in the abundance of these proteins could alter the free fraction and distribution of ertapenem. Whether such changes meaningfully affect ertapenem penetration into HS lesions or alter local immune signaling has not been directly demonstrated. HS is increasingly recognized as a systemic inflammatory disease supported by elevations in circulating IL-6, acute-phase reactants, and other inflammatory mediators that correlate with disease severity across independent patient cohorts [38,39,40,41]. For example, fibrinogen levels are elevated in patients with HS and correlate with disease severity, reflecting systemic inflammatory activation and vascular remodeling associated with chronic disease [32]. The same study demonstrated increased circulating angiopoietin-like protein 2 (ANGPTL2), an inflammatory mediator capable of activating NF-κB signaling and promoting expression of cytokines such as TNF-α and IL-6 [32]. α1-Antitrypsin (SERPINA1) is another ertapenem-binding plasma protein identified through compound-centric proteomic analysis, demonstrating measurable affinity for the drug alongside fibrinogen, immunoglobulin G, and other circulating proteins that can collectively sequester ertapenem in plasma [31]. Moreover, some ertapenem-binding proteins show direct correlation with HS severity. Haptoglobin concentrations are significantly increased in patients with HS and independently predict disease severity in prospective analyses [33]. Similarly, serum immunoglobulin G levels rise with increasing HS severity, suggesting enhanced adaptive immune activation and potential immune complex formation in advanced disease [34]. Together, these findings illustrate that many proteins capable of binding ertapenem are enriched within the inflammatory profile characteristic of HS. However, it remains unclear whether local or circulating increases in these proteins meaningfully alter ertapenem activity, and several of the reported biomarker associations are derived from a limited number of cohorts and await independent replication.

The overlap between ertapenem’s plasma binding partners and proteins elevated in HS suggests a potential link between inflammatory physiology and drug activity. Increased concentration of acute-phase proteins such as fibrinogen, haptoglobin, and immunoglobulins could alter ertapenem distribution, prolong systemic exposure, and modify the amount of free drug reaching inflamed tissues. Because many of these proteins accumulate within sites of chronic inflammation, altered protein binding may also influence how ertapenem penetrates HS lesions and the degree to which immune cells are exposed to both microbial products and antibiotic molecules within the local tissue environment. However, no studies have directly demonstrated that these alterations change ertapenem concentrations within HS lesions, and this remains a pharmacologic hypothesis that requires direct testing in patients with HS.

Changes in local drug availability may indirectly influence immune signaling within HS lesions, especially in macrophage- and neutrophil-rich inflammatory infiltrates that characterize active disease. These interactions provide a potential explanation for the clinical observations that ertapenem can produce rapid symptomatic improvement in severe HS despite the complex and polymicrobial nature of the disease. Altered protein binding within an inflammatory environment may influence drug distribution, half-life, and tissue penetration while also modifying immune signaling beyond antimicrobial activity alone. Although this concept remains largely hypothetical, it highlights an underexplored intersection between pharmacology and immunology that may help explain the distinctive clinical behavior of ertapenem in HS.

Limited ertapenem-specific experimental evidence also suggests that the drug may influence innate immune signaling, as it stimulates IL-1β release from monocytes even in the absence of bacteria such as *Staphylococcus aureus* [36]. This suggests that ertapenem possesses an intrinsic capacity to activate innate immune signaling, although the mechanism remains incompletely understood. When ertapenem is used in combination with cefazolin, IL-1β signaling is further amplified, particularly in the clearance of methicillin-susceptible *Staphylococcus aureus* (MSSA) bacteremia [36]. This suggests that cefazolin and ertapenem may act as immune modulators, potentially contributing to their strong clinical effectiveness in persistent MSSA bacteremia. However, the significance of increased IL-1β signaling in HS is unclear. Transient IL-1β release may enhance antimicrobial clearance during acute infection, but persistent or dysregulated IL-1β signaling can promote neutrophil recruitment and chronic inflammation [60]. Indeed, IL-1α- and IL-1β-targeted biologics have been evaluated as treatments for HS [61]. Furthermore, because the cell population examined consisted of healthy donor monocytes rather than monocytes derived from patients with HS, it is unclear whether an altered innate immune function would produce the same IL-1β response to ertapenem exposure. Therefore, ertapenem-induced IL-1β release may reflect a context-dependent immunomodulatory effect that supports antimicrobial defense during acute infection but could have different, or potentially opposing, consequences within the chronically inflamed microenvironment of HS lesions. In future studies, more meaningful results may be obtained by examining broader cytokine responses in normal monocytes as well as those isolated directly from patients with HS before and after ertapenem treatment. Additionally, to translate these findings to the nuances of HS, it would be beneficial to stratify results by Hurley stage and correlate them with clinical outcomes to determine whether ertapenem’s immunomodulatory effect is preserved in normal and pathologic monocytes.

## 5. Discussion

The clinical and mechanistic evidence suggests that ertapenem may benefit severe HS through effects that extend beyond antimicrobial activity alone. Across clinical studies, intravenous ertapenem has been associated with rapid reductions in pain, drainage, and overall disease burden, often with improvements in quality of life and inflammatory markers [13,16,21,24]. However, these clinical findings should be interpreted cautiously. As summarized in Table 1, the available studies are predominantly retrospective, single-center, and uncontrolled, with substantial heterogeneity in treatment duration and concurrent therapies. Because many patients received biologics, additional antibiotics, hormonal therapies, or surgery alongside ertapenem, the magnitude of improvement attributable specifically to ertapenem cannot be easily determined from the current literature. While these outcomes likely reflect partial suppression of polymicrobial colonization within HS lesions, their rapid onset raises the possibility that ertapenem may also influence host inflammatory signaling. Mechanistic studies demonstrate that carbapenems can affect cytokine signaling, endotoxin release, macrophage behavior, and immune-cell activation [2,27,28,29,30,31,32,33,34,36]. Clinically uninvolved skin in HS patients also remains microbiologically distinct from skin of healthy individuals, and whether ertapenem meaningfully alters this area, as opposed to acting primarily within active lesions, remains unknown [8]. However, clinical relapse after discontinuation of therapy is common in the absence of consolidation therapy, biologics, surgery, or additional systemic treatment. Thus, ertapenem may transiently reduce inflammatory burden rather than fully correcting the underlying immunologic and structural drivers of HS. This evidence positions ertapenem as a therapy that can stabilize severe disease while longer-term interventions are pursued.

Taken together, we propose that ertapenem should not be viewed solely as an antimicrobial agent in HS, nor as a conventional anti-inflammatory drug acting through a single immune pathway. Rather, its clinical effects may arise from the convergence of several processes: reduction in selected microbial populations, alteration of inflammatory signals generated during bacterial killing, and direct or indirect modulation of neutrophil- and macrophage-driven cytokine responses. In our view, the rapid improvement observed in severe HS despite incomplete or inconsistent suppression of some anaerobic taxa supports a model in which antimicrobial and host-directed effects occur simultaneously. The relative contribution of each mechanism is likely to depend on the microbial composition, inflammatory state, and stage of individual lesions.

This stabilizing effect has led to the use of ertapenem as a bridge to surgery or biologic therapy and as adjunctive treatment for patients with refractory disease requiring temporary disease control. These potential benefits, however, must be considered alongside important antimicrobial stewardship concerns and adverse events. Reported adverse events include Clostridium difficile infection, diarrhea, and PICC-line-associated deep vein thrombosis [24]. As a broad-spectrum carbapenem, ertapenem carries risks related to antimicrobial resistance, particularly in a chronic disease that may require repeated treatment courses. Its intravenous administration also introduces logistical burdens and safety considerations, including the need for PICC line placement and risk of catheter-related complications. These barriers may disproportionately affect patients already facing delays in HS diagnosis and treatment or limited access to dermatologic specialty care [62]. Since HS disproportionately affects individuals with skin of color and populations with structural barriers to healthcare access, equitable delivery of complex infusion-based therapies remains an important consideration in clinical decision-making [63]. Ertapenem is best viewed as a targeted intervention for patients with severe, refractory HS in whom short-term disease stabilization justifies the risks and logistical challenges associated with carbapenem therapy.

## 6. Methods

A structured literature search was conducted to identify studies examining the clinical use, antimicrobial activity, pharmacologic properties, and potential immunomodulatory effects of ertapenem and other carbapenems, with particular emphasis on HS. PubMed/MEDLINE was searched from database inception through 1 May 2026. During manuscript revision, a targeted update through 1 September 2026 was performed to identify newly published and reviewer-requested literature relevant to the scope of the review. Google Scholar was also reviewed to identify additional relevant publications. Search terms were used individually and in combination, including: “hidradenitis suppurativa,” “acne inversa,” “ertapenem,” “carbapenem,” “meropenem,” “imipenem,” “doripenem,” “antibiotic therapy,” “antimicrobial activity,” “immunomodulation,” “immune response,” “cytokine,” “interleukin,” “TNF,” “NF-kappa B,” “neutrophil,” “macrophage,” “endotoxin,” “lipopolysaccharide,” “plasma protein binding,” and “pharmacokinetics.” Eligible publications included clinical trials, cohort studies, case series, case reports, clinical guidelines, microbiologic studies, pharmacologic studies, and in vitro or in vivo mechanistic studies relevant to ertapenem or carbapenem-associated immune effects. Studies were included when they addressed at least one of the following: ertapenem treatment outcomes in HS, the HS lesional microbiome, antimicrobial or anti-biofilm activity, modulation of inflammatory signaling or innate immune-cell function, endotoxin release, or ertapenem pharmacokinetics and plasma protein binding.

Articles were excluded when they did not address HS, ertapenem, carbapenems, or a mechanism directly relevant to the scope of the review; lacked sufficient methodological or outcome information; were available only as abstracts; or were not published in English. Titles and abstracts were screened for relevance, followed by full-text assessment of potentially eligible publications. Because of substantial heterogeneity in study design, patient populations, experimental systems, treatment duration, and reported outcomes, the evidence was synthesized narratively rather than through meta-analysis.

## 7. Conclusions

Intravenous ertapenem has emerged as a valuable therapeutic option for patients with severe, refractory HS. Current evidence supports its role as a short-term rescue or bridge therapy rather than a definitive long-term treatment strategy. Clinical studies consistently demonstrate meaningful improvements in disease activity and quality of life, yet relapse after discontinuation remains common, highlighting the chronic inflammatory and structural drivers of advanced HS. While these benefits have traditionally been attributed to antimicrobial effects against the polymicrobial flora of HS lesions, emerging mechanistic evidence suggests that carbapenems may also influence host inflammatory pathways through effects on cytokine signaling, endotoxin release, immune cell activation, and protein-binding dynamics. This is an unproven but biologically plausible hypothesis warranting direct investigation in HS-specific models. Ertapenem’s broad antimicrobial spectrum and intravenous administration necessitate careful consideration of antimicrobial stewardship, emphasizing its most appropriate use as a targeted, short-term intervention in carefully selected patients.

Future research should focus on clarifying the immunologic effects of ertapenem directly within the HS disease environment. Prospective studies examining immune and cytokine profiles before and after ertapenem therapy could help determine whether clinical responses are accompanied by reproducible changes in inflammatory signaling pathways. In particular, evaluating mediators such as TNF-α, IL-1β, and IL-6, as well as macrophage- and neutrophil-associated inflammatory signatures, may provide insight into how carbapenems interact with the immune architecture of HS lesions. Comparative investigations across carbapenem antibiotics may also help determine whether the clinical behavior observed with ertapenem reflects drug-specific pharmacologic properties or broader class effects. The development of stewardship-informed treatment frameworks that clarify patient selection, treatment duration, and integration with surgery or biologic therapy will be essential for balancing the potential benefits of ertapenem with the long-term risks of broad-spectrum antimicrobial use.

## Figures and Tables

**Table 1:** Clinical Summary Report on Use of Ertapenem.

Study	Year	Number of Patients	Ertapenem Dose and Duration	Main Outcomes	Key Limitations
Join-Lambert [14]	2016	30	1 g daily IV for 6 weeks, then combined with consolidation triple antibiotic therapy for 6 months	Patients with Hurley stage I-II HS reached clinical remission. Patients with Hurley stage III reported major improvement in quality of life. Pain: Median pain score dropped from 6 to 0. Purulent Drainage: Median score dropped from 8 to 2. Sartorius score: dropped from a median of 49.5 to 19.0 after ertapenem and further to 12.0 at 6 months in compliant patients. QoL (daily handicap): Median score dropped from 8 to 3.	5 patients lost to follow-up. This was a retrospective study and was conducted under a monocentric design, thus making it susceptible to selection bias and limited generalizability. Lack of a control group.
Hamzavi [21]	2016	12	1 g daily for 62 days	QoL: 100% reported improvement. Drainage: 100% reported decreased drainage. Pain: 60% reported decreased pain.	Telephone interviews used for patient-reported outcomes; subject to recall bias. A limited sample size and a single-center population limit the generalizability of the findings.
Braunberger [13]	2018	36 included in chart review, 28 consented to follow-up interview	1 g daily IV, ranging from 8–128 days in tandem with benzoyl peroxide wash, prednisone, systemic antibiotics, biologics, spironolactone, topical silver nitrate, or nystatin powder	Of the 28 follow-up patients, 24 (85.7%) reported moderate or significant improvements in quality of life. 23 (82.14%) patients reported moderate or significant improvement in pain. 25 (89.29%) reported drainage improvement. 21 (75.0%) willing to undergo treatment again.	This was a retrospective study subject and was conducted under a monocentric design, thus making it susceptible to selection bias and limited generalizability. Incongruent follow-up intervals.
Nosrati [16]	2024	98	1 g IV for 12–16 weeks, topical antibiotics, antiandrogens, and biologics continued	PGA improved from 3.9 to 2.7. Pain decreased from 4.2 to 1.8. Clinically observed drainage reduction. 98 patients, 80% had medium-high satisfaction.	The single-center population limits the generalizability of the findings. Telephone interviews used to assess patient-reported satisfaction outcomes are subject to recall bias.
Ramesh [24]	2017	23	Daily IV, median 8 weeks	100% response rate within 2 weeks. All subjects relapsed. 3 patients who relapsed back to Hurley Stage III restarted Ertapenem after 3 months and had a successful response.	Conducted in a single center, limiting the generalizability of outcomes. Significant adverse events in eight patients following ertapenem therapy, including Clostridium difficile infection, diarrhea, and PICC-line-related deep vein thrombosis.

**Table 3:** Ertapenem-Binding Plasma Proteins with Relevance to Hidradenitis Suppurativa.

Protein	Normal Function	Relationship to Inflammation	Established Findings and Relevance to HS	Found in Tissue or Blood?
Haptoglobin	Acute-phase protein produced by the liver; binds free hemoglobin to limit oxidative damage; antibacterial	Upregulated during systemic inflammation; links obesity to chronic inflammation; Hp 2-2 phenotype associated with elevated TNF-α and IL-6 [42]; higher serum levels correlate with hyperinsulinemia [43]	Serum haptoglobin is the sole independent predictor of severe HS (Hurley III) by logistic regression (OR 1.011; *p* < 0.001). Levels rise stepwise across Hurley stages I → III and correlate with mHSS, SAHS, and DLQI. Haptoglobin also predicts risk of metabolic complications. Levels decreased post-excisional Surgery [44]. Haptoglobin is an ertapenem-binding plasma protein [37].	Blood (serum)
Immunoglobulin G (IgG)	Predominant antibody class mediating humoral immunity; activates complement via classical pathway; mediates antibody-dependent cellular cytotoxicity	IgG immune complex deposition sustains tissue inflammation; activates complement cascade; IgG and autoantibody accumulation in dermis increase with HS severity [45,46]	Serum IgG is an independent marker of HS severity (IHS4 and Hurley staging) in all adjusted models. AUC 0.90 (Hurley) and 0.79 (IHS4) for severe disease. Cut-off of 13.4 g/L carries 8-fold relative risk of Hurley stage III [46]. IgG immune complexes and complement deposits found in HS lesional skin [47].	Both blood (serum) and tissue
Fibrinogen	Coagulation factor I; converted by thrombin into fibrin during clot formation; inhibits plasminogen to block fibrinolysis	Acute-phase reactant; elevated in systemic inflammation; fibrinogen levels correlate with CRP and inflammatory markers; pro-coagulant environment links to vascular dysfunction [48]	Serum fibrinogen significantly elevated in HS patients vs. controls (310.0 vs. 266.5 mg/dL; *p* < 0.001). Correlates with Angiopoietin-like protein 2 levels and chronic inflammatory burden in HS [49]. Highest ertapenem-binding affinity among plasma proteins in SPR studies [37].	Blood (plasma/serum)
α-1-Antitrypsin (SERPINA1)	Serine protease inhibitor (serpin); neutralizes neutrophil elastase; tissue protective; anti-inflammatory role in lung and other tissues	Acute-phase protein; upregulated during inflammation [50]; SERPINA1 gene found upregulated in HS lesional skin transcriptomics (meta-analysis) [51]	SERPINA1 mRNA is upregulated in HS lesioned skin [51]. Binds ertapenem and reduces its antimicrobial activity in vitro [37].	Both tissue and blood
Transferrin	Iron-binding glycoprotein; transports iron in the bloodstream; regulates iron homeostasis	Negative acute-phase reactant (levels fall in inflammation); iron sequestration limits bacterial growth; iron dysregulation contributes to oxidative stress in inflammatory diseases [52]	Identified as an ertapenem-binding plasma protein [37]. Transferrin studied as a potential marker for metabolic complications alongside haptoglobin [44].	Blood (plasma)
Hemopexin	Plasma glycoprotein with very high affinity for heme; scavenges free heme released by hemoglobin turnover; protects against oxidative damage; supports iron homeostasis	Anti-inflammatory via heme scavenging [53]; limits heme-driven oxidative stress and TLR4 activation [54]	Identified as one of the four highest-affinity ertapenem-binding proteins in plasma proteomics and SPR (fibrinogen ≫ haptoglobin > IgG ≥ hemopexin) [37]. Potentially relevant to HS because oxidative stress is increased in patients with HS [55].	Blood (plasma)
Complement C4	Component of the classical and lectin complement pathways; forms part of the membrane attack complex to clear pathogens; bridges innate and adaptive immunity	Complement cascade activation is a prominent pathway in HS lesional skin [56]; complement dysregulation and IgG-mediated complement activation sustain inflammation [47]; upregulated complement genes found in HS transcriptomics [51]	Complement cascade is one of the most enriched pathways in HS lesional skin [51]. IgG hexamers assembled at cell surfaces activate complement via the classical pathway, and this mechanism is implicated in HS pathogenesis. Binds ertapenem and reduces its antimicrobial activity in vitro [37].	Both tissue and blood
Human Serum Albumin (HSA)	Most abundant plasma protein; carrier of hormones, fatty acids, bilirubin, and drugs; buffers pH; maintains oncotic pressure	Low albumin associated with poor outcomes [57]; limited direct pro-inflammatory role	HSA binds ertapenem at site 2 (nondelipidated form) [37]. Although HSA alone contributes modestly to ertapenem sequestration, it acts synergistically in protein cocktails. Not established as an independent severity biomarker in HS, but serum albumin can be affected by chronic inflammatory burden.	Blood (plasma)
Fibronectin	Extracellular matrix (ECM) glycoprotein; involved in cell adhesion, migration, wound healing, blood clotting, and tissue remodeling; binds integrins and collagen	Elevated in inflammatory and fibrotic conditions; involved in ECM remodeling [58]; ECM organization is a highly enriched pathway in HS (FDR 2.4 × 10^−14^ in meta-analysis) [51]; MMP-mediated ECM degradation is a key HS pathogenic mechanism [59]	ECM organization and collagen degradation pathways are prominently upregulated in HS lesional skin transcriptomics [51,59]. Fibronectin binds ertapenem and modestly reduces its antimicrobial activity [37]. Its role in dermal tunneling, scarring, and tissue remodeling in HS is biologically plausible.	Both tissue and blood

## Data Availability

All data are available within cited primary articles.

## Abbreviations

The following abbreviations are used in this manuscript:

ANGPTL2	Angiopoietin-like protein 2
AUC	Area under the curve
CCL	C-C motif chemokine ligand
CRP	C-reactive protein
CRKP	Carbapenem-resistant Klebsiella pneumoniae
CSF	Colony-stimulating factor
CSKP	Carbapenem-susceptible Klebsiella pneumoniae
CXCL	C-X-C motif chemokine ligand
DLQI	Dermatology Life Quality Index
ECM	Extracellular matrix
ESBL	Extended-spectrum beta-lactamase
GM-CSF	Granulocyte-macrophage colony-stimulating factor
hPBMCs	Human peripheral blood mononuclear cells
HS	Hidradenitis suppurativa
IFN-γ	Interferon-gamma
IgG	Immunoglobulin G
IHS4	International Hidradenitis Suppurativa Severity Score System
IL	Interleukin
IV	Intravenous
LOX-1	Lectin-like oxidized LDL receptor-1
LPS	Lipopolysaccharide
LRTI	Lower respiratory tract infection
MCP-1	Monocyte chemoattractant protein-1
MDR	Multidrug-resistant
mHSS	Modified Hidradenitis Suppurativa Score
MMP	Matrix metalloproteinase
MSSA	Methicillin-susceptible *Staphylococcus aureus*
NF-κB	Nuclear factor kappa B
NGS	Next-generation sequencing
OMV	Outer membrane vesicle
PGA	Physician Global Assessment
PICC	Peripherally inserted central catheter
SAHS	Severity Assessment of Hidradenitis Suppurativa
SERPINA1	Serpin family A member 1
sIL-2R	Soluble interleukin-2 receptor
SPR	Surface plasmon resonance
TLR4	Toll-like receptor 4
TNF-α	Tumor necrosis factor-alpha
